# Genome Sequence Resource of Bacillus velezensis Strain ML61, a Potential Biocontrol Bacterium Isolated from the Rhizosphere of Bok Choy (Brassica rapa var. *chinensis*)

**DOI:** 10.1128/mra.01311-22

**Published:** 2023-03-14

**Authors:** Liang Ma, Tong Liu

**Affiliations:** a College of Advanced Agricultural Sciences, Zhejiang A&F University, Lin’an, Hangzhou, People’s Republic of China; b State Key Laboratory for Conservation and Utilization of Bio-Resources in Yunnan, Yunnan University, Kunming, Yunnan Province, People’s Republic of China; Indiana University, Bloomington

## Abstract

Bacillus velezensis is widely known as a biocontrol agent against various plant diseases. Here, we report on the genome sequence of Bacillus velezensis strain ML61, which was isolated from the rhizosphere of bok choy (Brassica rapa var. *chinensis*) in Hangzhou, China, in 2020.

## ANNOUNCEMENT

**FIG 1:**
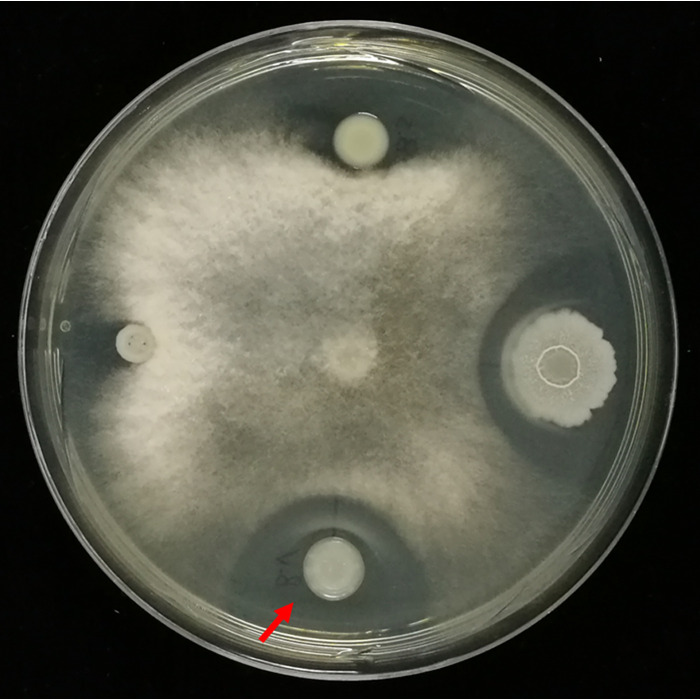
Inhibitory halos against Fusarium oxysporum of Bacillus velezensis ML61 (indicated by the red arrow). Bacterial colonies showing inhibition against F. oxysporum were isolated from rhizosphere soil of bok choy (Brassica rapa var. *chinensis*) by preliminary screening using an F. oxysporum-containing PDA plate. One microliter of bacterial suspension of each purified strain was inoculated onto the PDA plate, which was then inoculated with 5 μL of an F. oxysporum spore suspension (10^7^/mL) at the center. The image was taken after incubation for 4 days at 30°C.

Bacillus velezensis is well known for its ability to suppress microbial pathogens and promote plant growth (1, 2). Rhizosphere microorganisms can be exploited to promote plant growth and protect plants from pathogen attack (3). In this study, rhizosphere soil of bok choy (Brassica rapa var. *chinensis*), a common and important leafy vegetable in China (4), was collected and dissolved in sterile pure water. The suspension was diluted and spread onto potato dextrose agar (PDA) plates, which were seeded with Fusarium oxysporum spores (10^6^/mL, final concentration). After incubation for 4 days at 26°C, emergent bacterial colonies showing inhibitory halos were isolated and further purified by streaking onto a Luria-Bertani (LB) agar plate. Among them, a colony exhibiting a significant inhibitory halo was identified as Bacillus velezensis by analyzing its 16S ribosomal DNA sequence using BLASTn searches in the NCBI databases (5, 6), and we named this bacterial strain Bacillus velezensis ML61 (Fig. 1).

For genome sequencing, strain ML61 was propagated in LB liquid medium on a rotary shaker at 30°C for 24 h. Genomic DNA for PacBio sequencing was extracted using the Qiagen Genomic-tip 20/G kit. After that, the genomic DNA was fragmented using a Covaris g-TUBE device and concentrated using AMPure PB magnetic beads. Following DNA damage repair and end repair, the adapters were ligated. The treated DNA was purified and size selected using the BluePippin system (Sage Science) with cutoffs of 8 to 20 kb. The constructed library was bound with primers and polymerases using a PacBio binding kit. The final reaction products were sequenced using the Sequel II system (PacBio) with the Hi-Fi sequencing method, since it yields accurate long-read sequencing data sets (7, 8). The circular consensus sequencing (CCS) analysis workflow (SMRT Link software; PacBio) was used to trim adapters and compute consensus sequences from multiple passes around a single circularized DNA molecule. After filtering the low-quality and short reads (<2,000 bp), PacBio sequencing yielded 38,646 subreads, including 374,544,534 bp, with an *N*_50_ read length of 10,243 bp. The filtered subreads were assembled using Canu v1.5 software (9), and Circlator v1.5.5 was then used to circularize the genome assemblies (10). In the meantime, bacterial genomic DNA was extracted using the cetyltrimethylammonium bromide method (11), and a library was prepared using the Hieff next-generation sequencing (NGS) OnePot Pro DNA library prep kit for Illumina (Yeasen Biotech, Shanghai, China). Sequencing was performed on the Illumina NovaSeq 6000 platform with 2 × 150-bp paired-end sequencing. Adapters were removed from the sequence reads and low-quality reads were filtered using fastp (parameters, -q 10 -u 50 -y -g -Y 10 -e 20 -l 100 -b 150 -B 150). For quality control, the reads were filtered when more than 50% of bases had a Phred score lower than 10 or a read length of less than 100 bp. Illumina sequencing yielded a total of 1,274,611,406 clean bases with a Q30 value of 93.35%, and these data were used to correct the PacBio genome sequence using Pilon v1.22 (12).

A polished gapless circular chromosome of 4,035,016 bp with a GC content of 46.56% was finalized. Genome annotation was performed using the NCBI Prokaryotic Genome Annotation Pipeline (PGAP) (13), and it predicted 3,804 protein-coding genes, 27 rRNA genes, 86 tRNA genes, and 5 noncoding RNAs (ncRNAs). Using antiSMASH v5.0.0 (14), 13 secondary metabolite biosynthetic gene clusters were predicted. The whole-genome sequencing data set will provide us with a deeper understanding of the molecular basis for ML61 as a beneficial microorganism.

### Data availability.

The genome sequence of *B. velezensis* ML61 has been deposited at GenBank under the accession number CP103769. The associated BioProject and BioSample accession numbers for this project are PRJNA873517 and SAMN30498090, respectively.

## References

[1] Gouda S, Das G, Sen SK, Shin HS, Patra JK. 2016. Endophytes: a treasure house of bioactive compounds of medicinal importance. Front Microbiol 7:1538. doi:10.3389/fmicb.2016.01538. 27746767 PMC5041141

[2] Alenezi FN, Slama HB, Bouket AC, Cherif-Silini H, Silini A, Luptakova L, Nowakowska JA, Oszako T, Belbahri L. 2021. Bacillus velezensis: a treasure house of bioactive compounds of medicinal, biocontrol and environmental importance. Forests 12:1714. doi:10.3390/f12121714.

[3] de Zelicourt A, Al-Yousif M, Hirt H. 2013. Rhizosphere microbes as essential partners for plant stress tolerance. Mol Plant 6:242–245. doi:10.1093/mp/sst028. 23475999

[4] Zhou J, Wang J, Tao Y, Wang Y, Liu L, Wang S, Li Y, Zheng Q, Chen H. 2021. Metabolic response of bok choy leaves under chromium pollution stress. Ecotoxicology 30:231–239. doi:10.1007/s10646-020-02344-8. 33483874

[5] Janda JM, Abbott SL. 2007. 16S rRNA gene sequencing for bacterial identification in the diagnostic laboratory: pluses, perils, and pitfalls. J Clin Microbiol 45:2761–2764. doi:10.1128/JCM.01228-07. 17626177 PMC2045242

[6] Patel JB. 2001. 16S rRNA gene sequencing for bacterial pathogen identification in the clinical laboratory. Mol Diagn 6:313–321. doi:10.1054/modi.2001.29158. 11774196

[7] Wenger AM, Peluso P, Rowell WJ, Chang PC, Hall RJ, Concepcion GT, Ebler J, Fungtammasan A, Kolesnikov A, Olson ND, Topfer A, Alonge M, Mahmoud M, Qian Y, Chin CS, Phillippy AM, Schatz MC, Myers G, DePristo MA, Ruan J, Marschall T, Sedlazeck FJ, Zook JM, Li H, Koren S, Carroll A, Rank DR, Hunkapiller MW. 2019. Accurate circular consensus long-read sequencing improves variant detection and assembly of a human genome. Nat Biotechnol 37:1155–1162. doi:10.1038/s41587-019-0217-9. 31406327 PMC6776680

[8] Hon T, Mars K, Young G, Tsai Y-C, Karalius JW, Landolin JM, Maurer N, Kudrna D, Hardigan MA, Steiner CC, Knapp SJ, Ware D, Shapiro B, Peluso P, Rank DR. 2020. Highly accurate long-read HiFi sequencing data for five complex genomes. Sci Data 7:399. doi:10.1038/s41597-020-00743-4. 33203859 PMC7673114

[9] Koren S, Walenz BP, Berlin K, Miller JR, Bergman NH, Phillippy AM. 2017. Canu: scalable and accurate long-read assembly via adaptive k-mer weighting and repeat separation. Genome Res 27:722–736. doi:10.1101/gr.215087.116. 28298431 PMC5411767

[10] Hunt M, Silva ND, Otto TD, Parkhill J, Keane JA, Harris SR. 2015. Circlator: automated circularization of genome assemblies using long sequencing reads. Genome Biol 16:294. doi:10.1186/s13059-015-0849-0. 26714481 PMC4699355

[11] Clarke JD. 2009. Cetyltrimethyl ammonium bromide (CTAB) DNA miniprep for plant DNA isolation. Cold Spring Harb Protoc 2009:pdb.prot5177. doi:10.1101/pdb.prot5177. 20147112

[12] Walker BJ, Abeel T, Shea T, Priest M, Abouelliel A, Sakthikumar S, Cuomo CA, Zeng Q, Wortman J, Young SK, Earl AM. 2014. Pilon: an integrated tool for comprehensive microbial variant detection and genome assembly improvement. PLoS One 9:e112963. doi:10.1371/journal.pone.0112963. 25409509 PMC4237348

[13] Tatusova T, DiCuccio M, Badretdin A, Chetvernin V, Nawrocki EP, Zaslavsky L, Lomsadze A, Pruitt KD, Borodovsky M, Ostell J. 2016. NCBI Prokaryotic Genome Annotation Pipeline. Nucleic Acids Res 44:6614–6624. doi:10.1093/nar/gkw569. 27342282 PMC5001611

[14] Blin K, Shaw S, Steinke K, Villebro R, Ziemert N, Lee SY, Medema MH, Weber T. 2019. antiSMASH 5.0: updates to the secondary metabolite genome mining pipeline. Nucleic Acids Res 47:W81–W87. doi:10.1093/nar/gkz310. 31032519 PMC6602434

